# Supplementary material for: Effectiveness of Volatiles Emitted by Streptomyces abikoensis TJGA-19 for Managing Litchi Downy Blight Disease
Source: Microorganisms. 2024 Jan 17;12(1):184. doi: 10.3390/microorganisms12010184 (PMC10818274; doi:10.3390/microorganisms12010184)
Supplement: Supplementary file 1 [file microorganisms-12-00184-s001.zip › microorganisms-2802567-supplementary.pdf]

Table S1. Pathogenic fungal strains used in the study

S. No	Plant Pathogen
01	*Peronophythora litchii*
02	*Phytophthora capsici*
03	*Phytophthora colocasiae*
04	*Pythium myriotylum*
05	*Alternaria alternata*
06	*Pyricularia grisea*
07	*Neoscytalidium dimidiatum*
08	*Lasiodiplodia theobromae*
09	*Fusarium graminearum*
10	*Fusarium oxysporum* f.sp. *Cucumerinum*
11	*Bipolaris cactivora*
12	*Colletotrichum musarum*
13	*Colletotrichum gloeosporioides*
14	*F. oxysporum* f. sp. *cubense* Race 4
15	*Fusarium oxysporum* f.sp. *cubense* Race 1
16	*Cladosporium caricinum*
17	*Botryosphaeria berengeriana* f.sp

Table S2: Determination the antifungal spectrum of *S. abikoensis* TJGA-19 volatiles

S. No	Plant Pathogen	Inhibition（%）
01	*Peronophythora litchii*	100.0±0.0a
02	*Pythium myriotylum*	100.0±0.0a
03	*Alternaria alternata*	100.0±0.0a
04	*Colletotrichum musarum*	100.0±0.0a
05	*Phytophthora capsici*	93.3±0.2b
06	*Phytophthora colocasiae*	82.4±0.4c
07	*Lasiodiplodia theobromae*	72.1±1.2d
08	*Cladosporium caricinum*	64.3±0.4e
09	*Neoscytalidium dimidiatum*	55.2±1.3f
10	*Bipolaris cactivora*	43.3±0.8g
11	*Pyricularia grisea*	35.2±0.2h
12	*Fusarium graminearum*	28.1±0.6i
13	*Colletotrichum gloeosporioides*	20.5±1.1j
14	*Fusarium oxysporum* f.sp.*cubense* Race 1	12.6±0.5k
15	*Fusarium oxysporum* f.sp.*cubense* Race 4	0.0±0.0l
16	*Fusarium oxysporum* f.sp.*cucumerinum*	0.0±0.0l
17	*Botryosphaeria berengeriana* f.sp.	0.0±0.0l
